# Obsessive symptoms as first alert of psychosis: Two cases report

**DOI:** 10.1192/j.eurpsy.2021.1625

**Published:** 2021-08-13

**Authors:** L. Carpio Garcia, C. Martín Villarroel, M. Sánchez Revuelta, J. Dominguez Cutanda, J. Matsuura, E. García

**Affiliations:** Psychiatry, COMPLEJO HOSPITALARIO DE TOLEDO, Toledo, Spain

**Keywords:** psychosis, obsessive compulsive symptoms

## Abstract

**Introduction:**

Concomitant presence of obsessive-compulsive symptoms(OCS)is relatively frequent in psychotic patients and there are different hypotheses trying to explain the origin of them as pathology evolution,comorbid disorder,defence mechanism,or even a medication side-effect,but it is difficult to make a precise evaluation of these symptoms and the mechanisms involved.SometimesOCS are the first manifestation toappear without any other areas affected,and psychotic disorder comes later with initial symptoms in that domain.

**Objectives:**

Evaluate the association between OCS and psychosis to document pathogenia of both entities.

**Methods:**

A bibliographic search was performed about this topic.We present two cases of patients that have been referred to our unit: A34year old man, a usual consumer of cannabis,who shows checking and organizing compulsions that interfere significantly with their life.Consumptions grew progressively until they became daily,trying to decrease partly this behaviour. He comes to an addiction unit where he achieves abstinence,but immediately shows an important functional-impairment, adding to the previous compulsions new ones,and also thought blocking,social retraction and personality change. He starts taking antidepressant and benzodiazepines to reduceOCS, and weeks later begins a manic episode with delusions as a bipolar-disorder debut. A29year old man, with a history of familiar obsessive personality,that begins to worry about physical appearance and starts compulsive behaviour focused on exercise preventing him from daily activities.No response to antidepressants, he started antipsychotics and develop referential-symptoms.

**Results:**

Both are atypical presentations of bipolar and schizophreniform disorders withOCS,where the beginning of treatment causes psychosis-symptoms not previously developed.

**Conclusions:**

Frequent doubts are what factors determine the eclosion.The triggers are not clear and neither the related-pathology.

**Disclosure:**

No significant relationships.

